# Impact of Right Heart Failure on Outcomes of Transcatheter Aortic Valve Implantation: Insights from the National Inpatient Sample

**DOI:** 10.3390/jcm14030841

**Published:** 2025-01-27

**Authors:** Sajog Kansakar, Waqas T. Qureshi, Nava Raj Sharma, Dhan Bahadur Shrestha, Jurgen Shtembari, Vijay Shetty, Norbert Moskovits, Khagendra Dahal, Jishanth Mattumpuram, Daniel H. Katz

**Affiliations:** 1 Department of Internal Medicine, Maimonides Medical Center, Brooklyn, NY 11219, USA; sajog.kansakar@gmail.com (S.K.); nasharma@maimo.org (N.R.S.); 2 Division of Cardiology, Department of Internal Medicine, Houston Methodist, Houston, TX 77030, USA; drwaqasqureshi@gmail.com; 3 Division of Cardiology, Department of Internal Medicine, Bassett Medical Center, 1 Atwell Rd., Cooperstown, NY 13326, USA; daniel.katz@bassett.org; 4 Division of Cardiology, Department of Internal Medicine, Carle Foundation Hospital, 611 W Park St., Urbana, IL 61801, USA; jshtembari@gmail.com; 5 Division of Cardiology, Department of Internal Medicine, Maimonides Medical Center, Brooklyn, NY 11219, USA; vshetty@maimo.org (V.S.); nmoskovits@maimo.org (N.M.); 6 Division of Structural Interventional Cardiology, Department of Internal Medicine, Hartford Hospital, 80 Seymour Street, Hartford, CT 06102, USA; khagenvikram@hotmail.com; 7 Division of Cardiology, Department of Internal Medicine, University of Louisville School of Medicine, 550 S Jackson St., Louisville, KY 40202, USA; jishanth.mattumpuram@louisville.edu

**Keywords:** right heart failure, transcatheter aortic valve implantation, TAVI, mortality

## Abstract

**Background:** There are limited data on the impact of right heart failure (RHF) on patients undergoing transcatheter aortic valve implantation (TAVI). We investigated the mortality, in-hospital complications, length of stay, and total hospitalization charges for TAVI admissions, with and without RHF. **Methods:** We analyzed the National Inpatient Sample data from 2018 to 2022. The International Classification of Diseases–Tenth Revision (ICD-10) codes were used to define the patient cohorts. Propensity score weighting was used to balance patient demographic, hospital-level, and comorbidity data. **Results:** From 2018 to 2022, there were 383,860 TAVI admissions, among which 1915 (0.50%) had the presence of RHF. Compared to patients without RHF, mortality was higher in patients with RHF (7.57% vs. 1.11%, *p* < 0.01). Similarly, acute kidney injury (37.10% vs. 8.56%, *p* < 0.01), respiratory failure (12.79% vs. 1.91%, *p* < 0.01), and use of mechanical circulatory support (11.48% vs. 0.83%, *p* < 0.01) was higher in the cohort with RHF. Median length of stay (7 days vs. 2 days, *p* < 0.01) and hospitalization charges ($257,239 vs. $180,501, *p* < 0.01) were higher in patients with RHF. **Conclusions**: In conclusion, we report that RHF is associated with increased mortality risk, complications, and resource utilization in patients undergoing TAVI. Right ventricular function should be a part of the evaluation for TAVI, given significantly elevated risks associated with its presence.

## 1. Introduction

Transcatheter aortic valve implantation (TAVI) has surpassed surgical aortic valve replacement (SAVR) as the predominant modality for aortic valve replacement, and over 70,000 TAVI procedures are performed annually in the US [1]. The number of TAVI procedures for aortic stenosis is rapidly increasing as its use expands from high-risk or ineligible patients to those with intermediate and even low surgical risk [2,3,4]. Short-term and long-term complications, such as bleeding, vascular complications, stroke, arrhythmia, and aortic regurgitation, still exist [5]. However, evolving TAVI technology, identification of clinical and imaging predictors, and refined case selection have allowed the indication to expand to many patients not included in initial clinical trials [6,7,8].

One of the novel patient populations in which the outcome of TAVI is not clear is patients with right heart failure (RHF). Right ventricle (RV) dysfunction is defined by an abnormal RV structure or function with or without clinical signs of RHF. RHF is a syndrome with clinical features of heart failure as a result of RV dysfunction [9]. The prevalence of RV dysfunction in patients with aortic stenosis ranges from 8.7% to 29.1%, yet studies on its impact on outcomes of TAVI have been inconclusive [10,11,12,13,14,15]. In terms of RHF, there is a lack of literature regarding the prevalence of RHF in patients with aortic stenosis undergoing TAVI as well as its impact on its outcomes.

Thus, we conducted a retrospective analysis using the National Inpatient Sample (NIS) database, encompassing a large cohort of TAVI patients from 2018 to 2022. Our study aimed to investigate the association between RHF and in-hospital outcomes following TAVI, including mortality and post-procedural complications. We also examined the impact of RHF on length of stay and total hospitalization charges after TAVI.

## 2. Materials and Methods

We performed a retrospective cohort study using the National Inpatient Sample (NIS) database. We utilized the NIS dataset from the years 2018 and 2022. The NIS is sponsored by the Agency for Healthcare Cost and Utilization Project (AHRQ) [16]. The database is the largest publicly available, all-payer database of hospitalizations in the United States that represents a 20% stratified random sample of all hospital discharges from over 97% of hospitals participating in the Healthcare Cost and Utilization Project (HCUP). It contains clinical and resource utilization data on 5–8 million discharges annually from 1000 hospitals in 45 states. Institutional Review Board approval was not required for this study due to the de-identified nature of the database.

We identified our study population using the International Classification of Diseases–Tenth Revision (ICD-10) diagnostic and procedural codes. We sampled the NIS database to identify all admissions for adults (age ≥ 18 years) who underwent TAVI using ICD-10 PCS codes 02RF3JZ, 02RF38Z, 02RF3KZ, 02RF37Z, 02RF3JH, 02RF38H, 02RF3KH, and 02RF37H. For RHF, we used ICD-10 CM codes I50810, I50812, I50813, I50814, and I5082. We excluded patients under 18 years of age and patients with missing data on outcomes, which was less than one percent of the included population. We identified patient and hospital-level variables, such as age, sex, race, median household income for the patient’s ZIP code, insurance, hospital bed size, hospital region, and hospital location/teaching status using NIS. We identified comorbidities using ICD-10 codes (Supplementary Table S1).

The primary outcome was in-hospital mortality. The secondary outcomes were placement of permanent pacemakers, utilization of mechanical circulatory support (extracorporeal membrane oxygenation, intra-aortic balloon pump, Impella), myocardial/pericardial complications (myocardial injury, hemopericardium, cardiac tamponade, pericardiocentesis, pericardial window), respiratory failure, acute kidney injury, vascular complications (injury, rupture, AV fistula, hematoma), bleeding complications, venous thromboembolism, length of stay, and total hospital charges. We identified complications using ICD-10 CM and PCS codes (Supplementary Table S1).

To account for the complex survey design, data were analyzed after considering the stratification and discharge weights as recommended by HCUP NIS guidelines. Descriptive statistics were presented as frequency with percentage for categorical variables and median with interquartile range (IQR) for continuous variables. Pearson’s chi-square test was used for categorical variables, and the Mann–Whitney U test was used for continuous variables.

Propensity score weighting was used to balance the differences in baseline characteristics between patients admitted for TAVI with and without RHF. The propensity score for each patient was estimated on the basis of a multivariable logistic regression model of the probability of RHF on the basis of patient demographics, hospital-level, and comorbidity data given in Table 1. Then we used inverse probability weighting to generate propensity score weights that were incorporated into the survey sample weights as the new weights for our analysis [17]. Standardized mean differences and distribution of propensity scores were assessed after propensity score weighting (Supplementary Figure S1 and Table S2). We performed univariate analysis before and after matching. Binary outcomes were analyzed using logistic regression, and continuous outcomes were analyzed using negative binomial regression because of the skewed nature of the data. The Cochran–Armitage trend test was used to test for significant trends. A 2-tailed *p*-value < 0.05 was considered statistically significant. Analysis was performed using STATA-MP, version 14.2 (StataCorp LLC., Lakeway Drive, TX, USA).

## 3. Results

A total of 383,860 TAVI admissions between 2018 and 2022 were identified and included in the study. The population consisted of 166,160 (43%) females and 327,025 (87%) white patients with a median age of 79 (73–85). There were 1915 (0.5%) patients with RHF. The majority of patients (89%) were under Medicare insurance. Large (69%), urban teaching hospitals (89%) represent the majority of the TAVI population. The detailed baseline characteristics of patients who underwent TAVI with and without RHF are given in Table 1. Patients with RHF who underwent TAVI were younger (77 years vs. 79 years, *p* < 0.01), had a higher median Charlson Comorbidity Index (4 vs. 3, *p* < 0.01), and had a higher burden of comorbidities, such as chronic kidney disease (49% vs. 32%, *p* < 0.01), chronic liver disease (14% vs. 4%, *p* < 0.01), coagulopathy (29% vs. 10%, *p* < 0.01), and atrial fibrillation (38% vs. 28%, *p* < 0.01). Patients with RHF were less likely to be hypertensive (86% vs. 90%, *p* = 0.01) and smokers (22% vs. 35%, *p* < 0.01). Admissions for TAVI in patients with RHF were less likely to be elective admissions (51% vs. 84%, *p* < 0.01).

The mortality rate for TAVI admissions was 1.12% among the entire population. The most common complications were acute kidney injury (8.70%), the need for a permanent pacemaker (8.14%), and the requirement for blood transfusion (5.11%). The median length of stay was two days, and the median hospitalization charge was $180,740. During TAVI admissions, 145 (7.57%) patients with RHF died, while 4160 (1.11%) patients without RHF died.

On univariable analysis prior to propensity score weighting, TAVI admissions in patients with RHF were associated with higher odds of mortality (OR 7.44, 95% CI 5.05–10.96, *p* < 0.01), the need for mechanical circulatory support (OR 15.49, 95% CI 11.09–21.63, *p* < 0.01), respiratory failure (OR 7.55, 95% CI 5.50–10.36, *p* < 0.01), acute kidney injury (AKI) (OR 6.30, 95% CI 5.16–7.68, *p* < 0.01), blood transfusion (OR 2.12, 95% CI 1.51–2.97, *p* < 0.01), and venous thromboembolism (VTE) (OR 5.15, 95% CI 2.73–9.73, *p* < 0.01). Lengthier hospital stays and high total hospitalization charges were also seen in TAVI admissions in patients with RHF. The detailed outcomes are given in Table 2.

After propensity score weighting, in TAVI admissions, RHF was associated with higher odds of mortality (OR 4.11, 95% CI 2.25–7.52, *p* < 0.01), the need for mechanical circulatory support (OR 5.92, 95%CI 3.61–9.72, *p* < 0.01), respiratory failure (OR 4.26, 95% CI 2.36–7.71, *p* < 0.01), and AKI (OR 2.10, 95% CI 1.54–2.86, *p* < 0.01). Lengthier hospital stay and high total hospitalization charges were also seen. The detailed outcomes are given in Table 3.

**Figure 1:**
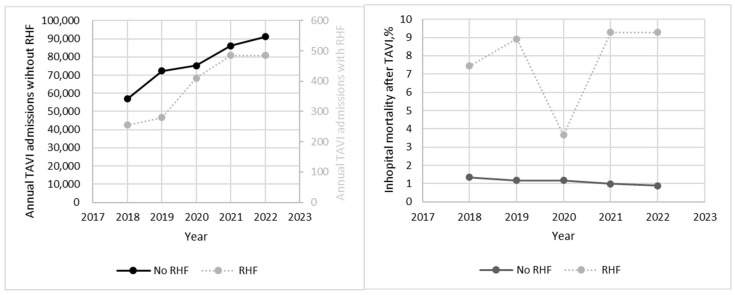
Trend in annual volume and in-hospital mortality for TAVI in patients with and without RHF. Abbreviations: TAVI, transcatheter aortic valve implantation; RHF, right heart failure.

In patients without RHF, the annual number of TAVIs increased from 56,900 to 91,140 between 2018 and 2022 (*p* trend = 0.04), while in patients with RHF, the annual number of TAVIs increased from 255 to 485 (*p* trend = 0.09). In patients without RHF, the in-hospital mortality for TAVI admission decreased from 1.34% to 0.88% (*p* trend = 0.13), while in patients with RHF, the in-hospital mortality for TAVI admission increased from 7.45% to 9.28% (*p* trend = 0.13). The trend for the annual number of procedures and in-hospital mortality is given in Figure 1.

## 4. Discussion

In this retrospective study using contemporary real-world population data from NIS, we investigated the effect of RHF on in-hospital outcomes during TAVI admission. The major findings of our study are as follows:

(1) Patients with RHF who underwent TAVI had a higher burden of comorbidities and had much higher in-hospital mortality (7.57% vs. 1.1%) compared to patients without RHF;
(2) Patients with RHF who underwent TAVI experienced more complications, such as acute kidney injury (37.10% vs. 8.56%), respiratory failure (12.79% vs. 1.91%), and the utilization of mechanical circulatory support (11.48% vs. 0.83%);
(3) Patients with RHF who underwent TAVI had lengthier hospital stays and higher total hospitalization charges.

In the developed world, AS is the most common valvular pathology, with a global prevalence of 9.4 million in 2019, primarily as a result of age-related calcific disease of the aortic valve [18]. In the past, severe AS was primarily treated surgically due to poor outcomes with medical management, with mortality approaching as low as 50% in 2 years [19]. SAVR remained the standard treatment strategy for severe AS; however, one-third of patients with severe AS were not deemed to be surgical candidates [20]. The Placement of Aortic Transcatheter Valve (PARTNER) and CoreValve series of trials have guided the evolution of TAVI from being the standard of care for patients with high surgical risk to a non-inferior to superior approach for moderate to low-risk patients [21]. As of 2017, TAVI overtook SAVR as the most common aortic valve replacement strategy for severe AS [1]. TAVI has a different profile of complications owing to the different nature of the procedure compared to SAVR. Notable complications include conduction abnormalities requiring a pacemaker, paravalvular leaks, stroke, and vascular complications [8].

With advances in TAVI technology and increased operator expertise, the incidence of inpatient complications after TAVI halved between 2012 and 2015 [22]. However, concern for mortality, complications, and its impact on healthcare costs exist [23]. Therefore, it is imperative to identify patients who are at risk of experiencing clinical adverse events following TAVI to refine selection [10,24]. Pre-procedural risk assessment tools, such as the STS score and EuroScore-II, are recommended by the American Heart Association/American College of Cardiology and European Society of Cardiology [25,26] before TAVI. The left ventricular function is integrated into both scores but not the RV function. Although conflicting results exist, several prior research studies have shown a significant association between RV dysfunction and increased mortality rates among TAVI patients [10,12,13,27]. However, to our knowledge, this is the first study to evaluate the outcome of TAVI in patients with a clinical diagnosis of RHF. Prior studies have evaluated the echocardiographic RV dysfunction and correlation with longer-term outcomes; our study is the largest and the first to evaluate in-hospital outcomes in patients with a clinical diagnosis of RHF [28,29,30].

A previous study using NIS data from 2010–2017 reported a mortality rate of 2.4% after TAVI [31]. The mortality rate for our study’s total population undergoing TAVI was 1.12%. These data suggest a trend toward decreasing mortality after TAVI, as reported in the current literature [22]. A higher mortality rate of 7.57% was seen in patients with RHF. A meta-analysis that included 3166 patients from eight studies calculated an odds ratio for mortality of 1.31 (95% CI 1.1–1.55, *p* = 0.002) in the presence of RV dysfunction [28]. However, a subgroup analysis of a PARTNER II trial noted no difference in one-year mortality regardless of the presence of RV dysfunction (adjusted hazard ratio 1.19, 95% CI 0.77–1.85, *p* = 0.44) [32]. Our study, which stratified patients based on a clinical diagnosis of RHF rather than imaging evidence of RV dysfunction, shows that the odds of mortality are much greater (OR 2.80, 95% CI 1.33–5.89, *p* = 0.01) in the clinical presence of RHF.

The possible underlying pathophysiological mechanism can be explained as follows [6,33,34]. Severe AS causes chronic pressure overload on the left ventricle, progressively leading to left-sided heart failure. Left-sided heart failure can lead to pulmonary venous congestion, which can lead to pulmonary capillary and arterial hypertension. This leads to chronic pressure overload on the RV, ultimately leading to RHF. TAVI can reduce the degree of left ventricular pressure overload from AS and possibly reverse the left ventricle hypertrophy but not the irreversible pulmonary vascular remodeling as well as the diffuse myocardial fibrosis that leads to persistent left ventricle diastolic dysfunction. Therefore, patients with RHF may not completely relieve pulmonary arterial hypertension after TAVI due to permanent LV and pulmonary vasculature remodeling. Furthermore, improved stroke volume after TAVI increases venous return to the right heart, which can cause volume overload on the failing RV. In our study, the higher mortality in patients with RHF is potentially driven by an increased incidence of life-threatening complications such as respiratory failure (12.79% vs. 1.91%) and potential cardiogenic shock (indicated by the use of mechanical circulatory support [11.48% vs. 0.83%]). These complications, along with acute renal injury (37.10% vs. 8.56%) can be explained by these hemodynamic changes after TAVI in patients with RHF. There were no differences in permanent pacemaker implantation, vascular, myocardial, or pericardial complications between the two cohorts. This is likely due to these adverse outcomes being less likely associated with hemodynamic differences between the two groups.

Our study also showed the economic burden associated with TAVI with comorbid RHF. Patients with RHF were hospitalized for an additional five days, and hospital charges were higher by $70,385 than those without RHF. This finding might be explained by the higher incidence of complications in this population group leading to a prolonged hospital stay and resource utilization.

Given the findings of our study, showing significantly elevated complications and in-hospital mortality in patients with RHF who underwent TAVI (7.57% vs. 1.11%), clinical and echocardiographic evaluation of RV function must be assessed during evaluation patients for TAVI as pre-procedural risk assessment tools, such STS score and EuroScore-II, do not address RV function. Operative mortality after SAVR is markedly increased in the presence of RV dysfunction, and TAVI may still be favored over SAVR in patients who undergo aortic valve replacement [35]. The outcome data from our study can be used to make an informed decision with a patient with RHF during discussion about the risks and benefits of TAVI. RHF patients undergoing TAVI may benefit from strict hemodynamic monitoring during peri-procedure for early identification and mitigation of hemodynamic collapse. Furthermore, given the much higher use of mechanical circulatory support in TAVI patients with RHF (11.48% vs. 0.83%), availability of such resources must be considered during the planning of TAVI in patients with RHF. Furthermore, careful monitoring of renal function and respiratory function is crucial given the markedly higher prevalence of these complications in the presence of RHF.

While our study provides valuable insights into the association between RHF and TAVI outcomes, several limitations should be acknowledged in our study. The retrospective nature of our analysis and reliance on administrative data introduce inherent biases and limitations associated with coding accuracy and data completeness. There might be subjective variation in clinicians’ clinical diagnoses of RHF for all the patients included in the database as RHF is a clinical diagnosis which includes clinical symptoms as well as imaging evidence of right ventricle dysfunction. The NIS database lacks long-term follow-up data beyond the in-hospital events, precluding our ability to assess outcomes beyond the index hospitalization. Furthermore, information such as clinical severity of RHF, or echocardiographic parameters of RV function are not available in the database, which may be important to evaluate how the degree of RHF might be correlated to mortality. Although we used propensity score weighting to adjust for differences in baseline characteristics between the groups, the RHF group had co-morbidities such as atrial fibrillation, chronic lung disease, chronic kidney disease, and coagulopathy, which could explain the differences in outcomes between the two groups. Furthermore, the impact of unmeasured confounders could not be determined. Future research should aim to address these limitations by incorporating more comprehensive clinical data and long-term follow-up assessments to provide a more nuanced understanding of the impact of RHF on TAVI outcomes. Despite these limitations, the study’s strength lies in the large sample size, the generalizability of our study compared to single-center studies, and the use of a reliable and extensively used database.

## 5. Conclusions

In conclusion, we report the real-world data outcomes from a large nation-wide database on the prevalence of RHF and its impact on in-hospital outcomes of patients who undergo TAVI. The presence of RHF is strongly correlated with worse in-hospital outcomes following TAVI. Patients with RHF undergoing TAVI are at increased risk of mortality, post-procedural complications, prolonged hospitalizations, and higher healthcare resource utilization. Our study adds to the growing evidence that clinical and echocardiographic evaluation of RV function must be assessed during the evaluation of patients for TAVI as routine pre-procedural risk assessment tools do not assess RV function.

## Figures and Tables

**Table 1:** Baseline characteristics of patients who underwent TAVI with and without RHF.

Baseline Characteristics, n (%)	Total	No RHF	RHF	*p* Value
	n = 383,860	n = 381,945	n = 1915	
Female	166,160 (43%)	165,465 (43%)	695 (36%)	<0.01
Age, years, median (IQR)	79 (73–85)	79 (73–85)	77 (68–84)	<0.01
White race	327,025 (87%)	325,465 (87%)	1560 (86%)	0.35
Charlson Comorbidity Index				<0.01
1.00	78,440 (20%)	78,195 (20%)	245 (13%)	
2.00	78,435 (20%)	78,115 (20%)	320 (17%)	
3.00 or more	196,860 (51%)	195,510 (51%)	1350 (71%)	
Median household income for patient’s ZIP				0.26
0–25th percentile	80,460 (21%)	80,100 (21%)	360 (19%)	
26th to 50th percentile (median)	97,895 (26%)	97,335 (26%)	560 (30%)	
51st to 75th percentile	100,850 (27%)	100,330 (27%)	520 (28%)	
76th to 100th percentile	99,595 (26%)	99,150 (26%)	445 (24%)	
Insurance				<0.01
Medicare	333,120 (89%)	331,615 (89%)	1505 (81%)	
Medicaid	6085 (2%)	5990 (2%)	95 (5%)	
Private insurance	33,870 (9%)	33,650 (9%)	220 (12%)	
Self-pay	1735 (0%)	1690 (0%)	45 (2%)	
Region of hospital				<0.01
Northeast	84,420 (22%)	84,135 (22%)	285 (15%)	
Midwest	90,025 (23%)	89,430 (23%)	595 (31%)	
South	130,850 (34%)	130,245 (34%)	605 (32%)	
West	78,630 (20%)	78,200 (20%)	430 (22%)	
Hospital bed size				0.03
Small	32,340 (8%)	32,225 (8%)	115 (6%)	
Medium	86,585 (23%)	86,240 (23%)	345 (18%)	
Large	265,000 (69%)	263,545 (69%)	1455 (76%)	
Location/teaching status of hospital				0.22
Rural	6150 (2%)	6105 (2%)	45 (2%)	
Urban nonteaching	34,525 (9%)	34,395 (9%)	130 (7%)	
Urban teaching	343,250 (89%)	341,510 (89%)	1740 (91%)	
Elective	322,430 (84%)	321,465 (84%)	965 (51%)	<0.01
Comorbidities				
Hypertension	345,740 (90%)	344,090 (90%)	1650 (86%)	0.01
Diabetes mellitus	144,460 (38%)	143,700 (38%)	760 (40%)	0.38
Chronic kidney disease	124,315 (32%)	123,385 (32%)	930 (49%)	<0.01
Peripheral vascular disease	76,625 (20%)	76,205 (20%)	420 (22%)	0.35
Coronary artery disease	259,615 (68%)	258,365 (68%)	1250 (65%)	0.32
Chronic lung disease	94,970 (25%)	94,425 (25%)	545 (28%)	0.09
Chronic liver disease	15,190 (4%)	14,920 (4%)	270 (14%)	<0.01
Obesity	85,115 (22%)	84,650 (22%)	465 (24%)	0.31
Smoker	134,250 (35%)	133,825 (35%)	425 (22%)	<0.01
Coagulopathy	37,665 (10%)	37,115 (10%)	550 (29%)	<0.01
Atrial fibrillation	107,005 (28%)	6270 (28%)	735 (38%)	<0.01

Abbreviations: TAVI, transcatheter aortic valve implantation; RHF, right heart failure.

**Table 2:** In-hospital outcomes in TAVI with and without RHF before propensity score weighting.

Outcome, n (%)	Total	No RHF	RHF	OR	95% CI	*p* Value
Mortality	4305 (1.12%)	4160 (1.11%)	145 (7.57%)	7.44	5.05–10.96	<0.01
Permanent pacemaker implantation	31,260 (8.14%)	31,075 (8.13%)	185 (9.66%)	1.21	0.83–1.76	0.33
Mechanical circulatory support *	3395 (0.88%)	3175 (0.83%)	220 (11.48%)	15.49	11.09–21.63	<0.01
Myocardial or pericardial complications **	4950 (1.29%)	4910 (1.28%)	40 (2.08%)	1.64	0.81–3.30	0.17
Respiratory failure	7530 (2.00%)	7285 (1.91%)	245 (12.79%)	7.55	5.50–10.36	<0.01
Acute kidney injury	33,405 (8.70%)	32,695 (8.56%)	710 (37.10%)	6.30	5.16–7.68	<0.01
Vascular complications ***	5450 (1.42%)	5415 (1.41%)	35 (1.83%)	1.29	0.62–2.70	0.49
Blood transfusion	19,620 (5.11%)	19,425 (5.08%)	195 (10.20%)	2.12	1.51–2.97	<0.01
Venous thromboembolism	2030 (0.50%)	1980 (0.50%)	50 (2.60%)	5.15	2.73–9.70	<0.01
Length of stay, days, median (IQR)	2 (1–3)	2 (1–3)	7 (2–15)	-		<0.01
Total charges, $, median (IQR)	180,740 (133,808– 264,586)	180,501 (133,702–264,007)	257,239 (171,467–411,012)	-		<0.01

* Extracorporeal membrane oxygenation, intra-aortic balloon pump, Impella. ** Myocardial injury, hemopericardium, cardiac tamponade, pericardiocentesis, pericardial window. *** Injury, rupture, AV fistula, hematoma. Abbreviations: TAVI, transcatheter aortic valve implantation; RHF, right heart failure; OR, odds ratio; 95% CI, 95% confidence interval; IQR, interquartile range; $ United States Dollar.

**Table 3:** In-hospital outcomes in TAVI with and without RHF after propensity score weighting.

Outcome, n (%)	OR	95% CI	*p* Value
Mortality	4.11	2.25–7.52	<0.01
Permanent pacemaker implantation	0.90	0.51–1.58	0.711
Mechanical circulatory support *	5.92	3.61–9.72	<0.01
Myocardial or pericardial complications **	1.12	0.44–2.83	0.811
Respiratory failure	4.26	2.36–7.71	<0.01
Acute kidney injury	2.10	1.54–2.86	<0.01
Vascular complications ***	0.84	0.20–3.47	0.813
Blood transfusion	1.00	0.59–1.68	0.995
Venous thromboembolism	1.78	0.80–3.97	0.157
Length of stay, days	-		<0.01
Total charges, $	-		<0.01

* Extracorporeal membrane oxygenation, intra-aortic balloon pump, Impella. ** Myocardial injury, hemopericardium, cardiac tamponade, pericardiocentesis, pericardial window. *** Injury, rupture, AV fistula, hematoma. Abbreviations: TAVI, transcatheter aortic valve implantation; RHF, right heart failure; OR, odds ratio; 95% CI, 95% confidence interval; $ United States Dollar.

## Data Availability

All data related to the study are presented in the study and Supplementary Files.

## Supplementary Materials

The following supporting information can be downloaded at https://www.mdpi.com/article/10.3390/jcm14030841/s1, Table S1: International Classification of Diseases, 10th revision, clinical modification/procedure coding system (ICD-10 CM/PCS) codes; Table S2. Table showing standardized mean difference between no RHF and RHF group before and after propensity score matching (PSM); Figure S1. Figure showing log propensity score vs. density before and after propensity score matching.
