# Supplementary material for: Impact of Right Heart Failure on Outcomes of Transcatheter Aortic Valve Implantation: Insights from the National Inpatient Sample
Source: J Clin Med. 2025 Jan 27;14(3):841. doi: 10.3390/jcm14030841 (PMC11818276; doi:10.3390/jcm14030841)

Supplementary Table S1. International Classification of Diseases,10th revision, clinical modification/procedure coding system (ICD-10 CM/PCS) codes

**Variable**		**ICD-10 code**	
TAVI/ TAVI		PCS	02RF38H 02RF38Z 02RF3KH 02RF3KZ
RHF		CM	I50810 I50812 I50813 I50814 I5082
Permanent pacemaker		PCS	0JH60PZ, 0JH63PZ 0JH80PZ 0JH604Z 0JH634Z 0JH804Z 0JH834Z 0JH605Z 0JH635Z 0JH805Z 0JH835Z 0JH606Z 0JH636Z 0JH806Z 0JH836Z 02HK4JZ 02HK3JZ 02HK0JZ 0JH607Z 0JH637Z 0JH807Z 0JH837Z 02HL4JZ 02HL3JZ 02HL0JZ 02H44JZ 02H43JZ 02H40JZ
IABP		PCS	5A02110 5A02210
PVAD		PCS	02HA3RZ 02HA3RS 02HA0RZ 02HA3QZ 02HA3RJ 5A0221D 5A0211D 5A02116 5A02216
ECMO		PCS	5A15223 5A1522F 5A1522G 5A1522H 5A15A2F 5A15A2G 5A15A2H
Myo/pericardial complications	Tamponade	CM	I314
	Hemopericardium	CM	I312
	Myocardial injury	CM	S261**A S26.9**A S260**A I9751
	Window/ centesis	PCS	0W9D00Z 0W9D0ZZ 0W9C00Z 0W9C0ZZ 0W9D30Z 0W9D3ZZ 0W9D40Z 0W9D4ZZ 0W9C30Z 0W9C3ZZ 0W9C40Z 0W9C4ZZ
Respiratory failure		CM	5A1955Z 5A1935Z 5A1945Z
		PCS	J95821 J9581 J9589
AKI		CM	N17*
Vascular comp	Injury	CM	S090***A S15***A S25***A S35***A S45***A S55***A S65***A S75***A S85***A
	Rupture	CM	I772 T8183XA
	AV fistula	CM	I770
	Hematoma	CM	I770 I97621 I97630 I97631 I97638
	Unspecified	CM	T817*

VTE	Lower extremity DVT	CM	I82401 I82402 I82403 I82409 I82411 I82412 I82413 I82419 I82421 I82422 I82423 I82429 I82431 I82432 I82433 I82439 I82441 I82442 I82443 I82449 I82451 I82452 I82453 I82459 I82461 I82462 I82463 I82469 I82491 I82492 I82493 I82499 I824Y1 I824Y2 I824Y3 I824Y9 I824Z1 I824Z2 I824Z3 I824Z9
	PE	CM	I2601 I2602 I2609 I2690 I2692 I2693 I2694 I2699
Hypertension		Elixhauser comorbidity mapping	
Diabetes mellitus		Elixhauser comorbidity mapping	
CKD		N18*, Z992, Z9115	
Peripheral vascular disease		Elixhauser comorbidity mapping	
Coronary artery disease		I251*, I252, I255, I256, I257*, I258*, I259	
Heart failure		Elixhauser comorbidity mapping	
Chronic lung disease		Elixhauser comorbidity mapping	
Obesity		Elixhauser comorbidity mapping	
Smoking		Z87891 F17200 F17203 F17201 F17208 F17209	
Malignancy		Elixhauser comorbidity mapping	
Coagulopathy		Elixhauser comorbidity mapping	
Atrial fibrillation		I480 I481 I482 I4891	

Supplementary Table S2 . Table showing standardized mean difference between no RHF and RHF group before and after propensity score matching (PSM)

Before PSM

	Mean in treated	Mean in Untreated	Standardised diff.
AGE	74.83	78.28	-0.339
FEMALE	0.37	0.44	-0.145
RACE2	0.14	0.13	0.057
charlsoncat	2.58	2.15	0.495
ZIPINC_QRTL	2.56	2.59	-0.030
insure	1.37	1.21	0.225
HOSP_REGION	2.61	2.52	0.088
HOSP_BEDSIZE	2.71	2.61	0.158
teaching	0.90	0.89	0.035
ELECTIVE	0.51	0.85	-0.773
HTN	0.86	0.90	-0.114
DM	0.39	0.37	0.025
CKD	0.49	0.32	0.351
PVD	0.23	0.20	0.072
CAD	0.65	0.68	-0.046
LUNG	0.29	0.25	0.090
LIVER	0.14	0.04	0.356
OBESE	0.23	0.22	0.029
SMOKE	0.22	0.35	-0.296
COAG	0.29	0.10	0.493
AFIB	0.39	0.28	0.238

After PSM

	Mean in treated	Mean in Untreated	Standardised diff.
AGE	80.17	78.27	0.187
FEMALE	0.53	0.44	0.173
RACE2	0.10	0.13	-0.071
charlsoncat	2.24	2.15	0.100
ZIPINC_QRTL	2.68	2.59	0.088
insure	1.14	1.21	-0.090
HOSP_REGION	2.53	2.52	0.010
HOSP_BEDSIZE	2.54	2.61	-0.115
teaching	0.90	0.89	0.014
ELECTIVE	0.86	0.84	0.029
HTN	0.94	0.90	0.134
DM	0.32	0.38	-0.119
CKD	0.33	0.33	0.003
PVD	0.19	0.20	-0.032
CAD	0.68	0.68	0.004
LUNG	0.25	0.25	0.019
LIVER	0.04	0.04	-0.015
OBESE	0.20	0.22	-0.058
SMOKE	0.34	0.35	-0.017
COAG	0.09	0.10	-0.031
AFIB	0.34	0.28	0.127

Supplementary Figure S1. Figure showing log propensity score vs density before and after propensity score matching

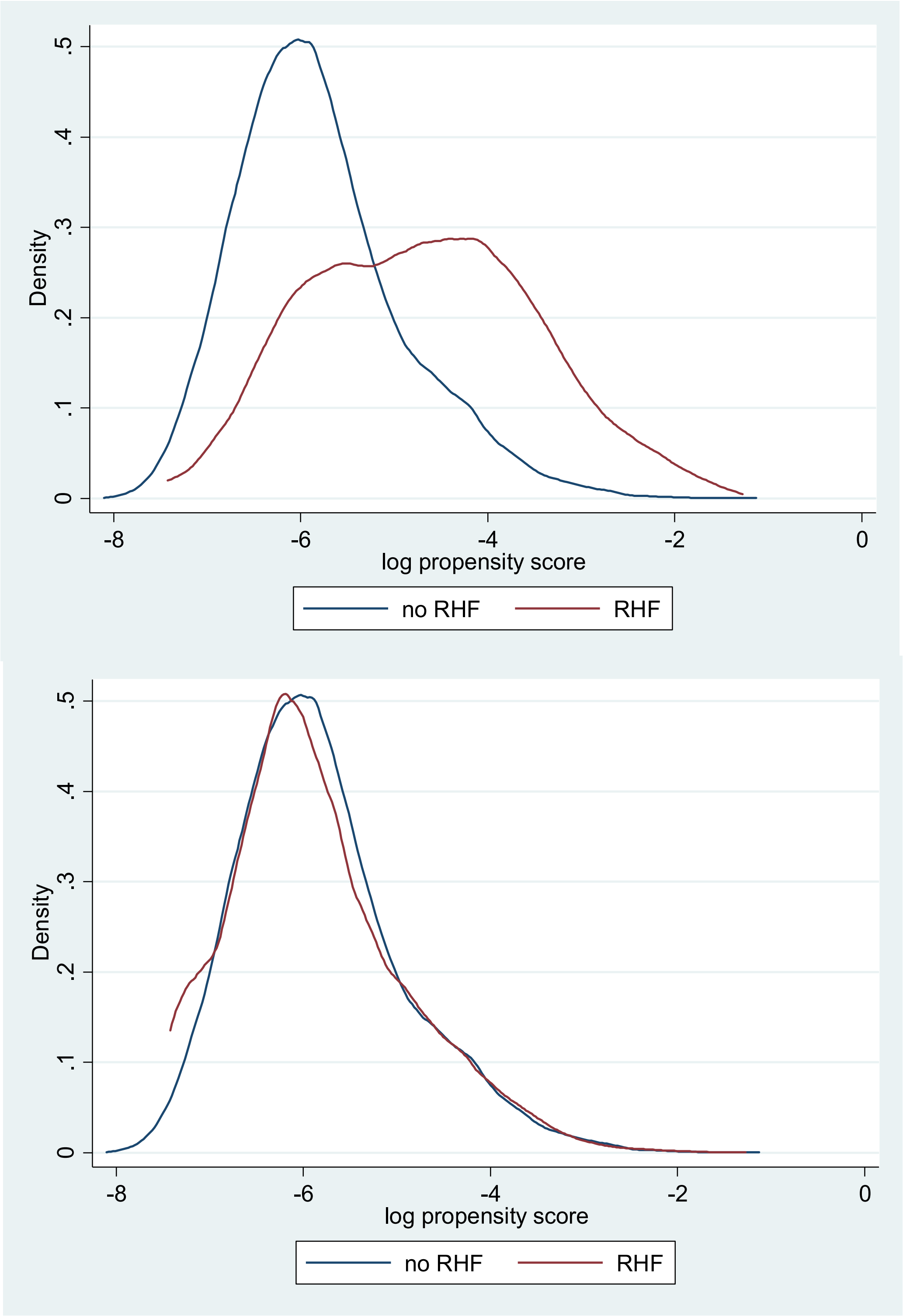

Supplement: Supplementary file 1 [file jcm-14-00841-s001.zip › jcm-3400193-supplementary.pdf]
